# Genetic Variations as Modifying Factors to Dietary Zinc Requirements—A Systematic Review

**DOI:** 10.3390/nu9020148

**Published:** 2017-02-17

**Authors:** Kaitlin J. Day, Melissa M. Adamski, Aimee L. Dordevic, Chiara Murgia

**Affiliations:** Department of Nutrition, Dietetics and Food, Monash University, Notting Hill VIC 3168, Australia; kjday1@student.monash.edu (K.J.D.); melissa.adamski@monash.edu (M.M.A.); aimee.dordevic@monash.edu (A.L.D.)

**Keywords:** zinc requirements, SNPs, nutrigenetics, nutritional genomics

## Abstract

Due to reduced cost and accessibility, the use of genetic testing has appealed to health professionals for personalising nutrition advice. However, translation of the evidence linking polymorphisms, dietary requirements, and pathology risk proves to be challenging for nutrition and dietetic practitioners. Zinc status and polymorphisms of genes coding for zinc-transporters have been associated with chronic diseases. The present study aimed to systematically review the literature to assess whether recommendations for zinc intake could be made according to genotype. Eighteen studies investigating 31 Single Nucleotide Polymorphisms (SNPs) in relation to zinc intake and/or status were identified. Five studies examined type 2 diabetes; zinc intake was found to interact independently with two polymorphisms in the zinc-transporter gene *SLC30A8* to affect glucose metabolism indicators. While the outcomes were statistically significant, the small size of the effect and lack of replication raises issues regarding translation into nutrition and dietetic practice. Two studies assessed the relationship of polymorphisms and cognitive performance; seven studies assessed the association between a range of outcomes linked to chronic conditions in aging population; two papers described the analysis of the genetic contribution in determining zinc concentration in human milk; and two papers assessed zinc concentration in plasma without linking to clinical outcomes. The data extracted confirmed a connection between genetics and zinc requirements, although the direction and magnitude of the dietary modification for carriers of specific genotypes could not be defined. This study highlights the need to summarise nutrigenetics studies to enable health professionals to translate scientific evidence into dietary recommendations.

## 1. Introduction

Genetic background can affect a person’s nutritional status. This may have the potential to modify an individual’s optimal nutrient requirements and risk of developing specific pathologic conditions [1]. Zinc (Zn) is an essential micronutrient that plays fundamental roles in several aspects of physiology, cellular metabolism, and gene expression [2]. In Australia, the current Recommended Dietary Intake (RDI) for Zn is 14 mg/day for men and 8 mg/day for women (19+ years), with an upper limit of 40 mg/day [3]. Similarly, in the US, the current Recommended Dietary Allowance for Zn is 11 mg/day for men and 8 mg/day for women (19+ years) [4]. Zn deficiency is the cause of a complex syndrome [5], more subtle and difficult to assess is marginal Zn deficiency; while currently not well-understood, it may be a contributing factor in chronic disease development [6]. Zn deficiency can arise from several causes including specific genetic background [7]. For example, mutations in the gene coding for the intestinal transporter *SLC39A4* cause the inherited disorder Acrodermatitis Enteropathica (AE), a condition caused by the inability to absorb Zn and resulting in systemic deficiency that is resolved with lifelong Zn supplementation (1 mg/kg/day body weight) [8].

Zn crosses biological membranes with the aid of specialized trans-membrane proteins. More than 20 proteins coordinate their activity to maintain systemic and cellular Zn homeostasis; in mammals, ZnT (coded by *SLC30A* genes) and ZIP (coded by *SLC39A* genes) were identified for this role. Intracellular Zn concentration is buffered by Metallothioneins (MTs), a class of proteins with high affinity for metals [9]. With the completion of the human genome sequence, all Zn transporter genes were identified and several common polymorphisms and rare mutations were found to be associated with human pathologies [6,10]. 

Insulin metabolism in the pancreatic β-cells requires Zn [11], and intracellular Zn directly acts on insulin receptors’ intracellular pathways. A Single Nucleotide Polymorphism (SNP) in the gene coding for the Zn transporter *SLC30A8* (rs13266634) has been identified as being associated with increasing the risk of developing type 2 diabetes (T2D); as such, modification of Zn requirements was hypothesized as a potential method to reduce the risk of T2D for rs13266634 carriers [12,13]. Another condition that is impacted by genetic polymorphisms in Zn transporters is mammary gland secretion of Zn in milk. Null mutations in genes coding for transporters expressed in mammary epithelium result in milk Zn concentrations that are insufficient to support the growth of babies, ultimately leading to poor infant health outcomes [8,14,15]. Zn status has been also associated with cognitive performance [16]. Genetic variations have been explored in cognitive studies and may also play a role in conditions such as Alzheimer’s Disease (AD) [17], a concept that is yet to be investigated in human studies [18]. Many studies have investigated genetic polymorphisms in regards to gene-nutrient interactions and nutrient status in the onset and progression of specific diseases. However, the question remains; is there sufficient evidence to make specific dietary and nutrition recommendations based on genotype? [19]. The aim of this paper was to systematically review the evidence to answer whether specific polymorphisms modify individual dietary Zn requirements and whether current evidence can inform clinical dietetic practice.

## 2. Materials and Methods

### 2.1. Search Methods

A literature search was conducted in October, 2015. The following search strategy was used to search Medline, Embase, and CENTRAL. This strategy was then adapted to search Web of Science, CINAHL and Scopus databases (S1). (See PRISMA checklist (S2)).

1. Zinc.ti,ab.
2. Zn.ti,ab.
3. Zinc/
4. Zinc compounds/
5. Finger*.ti,ab.
6. 1 OR 2 OR 3 OR 4
7. 6 NOT 5
8. (Polymorph* OR Allel* OR Genet* OR Geno* OR Gene OR Genes).ti,ab.
9. Exp Polymorphism, Genetic/
10. 8 OR 9
11. Exp Cation Transport Proteins/
12. (Zinc adj2 deficienc*).ti,ab.
13. (Zn adj2 deficienc*).ti,ab.
14. (Zinc adj2 transporter*).ti,ab.
15. (Zn adj2 transporter*).ti,ab.
16. 11 OR 12 OR 13 OR 14 OR 15
17. 7 AND 10 AND 16

### 2.2. Inclusion Criteria

Studies were included if they were conducted in mammals, in particular humans, mice, and rats, if they investigated polymorphisms within the population, if they modulated plasma Zn concentrations or investigated whether this interaction was modulated by Zn status. Study designs that were observational, case-control, and clinical trials were eligible for inclusion in this review.

### 2.3. Exclusion Criteria

Studies were excluded that were not written in English. Studies were also excluded that were examining type 1 diabetes, conducted before 1995 (when the first gene coding for a Zn transporter was identified [20]), single case studies, book chapters, editorial letters, conference proceedings, and reviews. 

### 2.4. Data Collection and Analysis

#### 2.4.1. Selection of Studies

Three reviewers (KD, MA, and CM) independently screened the titles and abstracts of the studies retrieved by the above search strategy. Full articles were then retrieved and were independently assessed by the same three reviewers for inclusion using the criteria outlined above. Conflicts were discussed and decided upon as a group. Reference lists of relevant studies and reviews were also manually searched.

#### 2.4.2. Data Extraction

An extraction table was developed to include information about study design, association of genetic variants with phenotypic traits, and other relevant outcomes. Three review authors (KD, MA, and CM) independently extracted the data into an extraction table which included zinc status method, genotyping method, polymorphisms identified, association between SNP, zinc status and biomarkers, association between SNP, zinc status, and disease state. The data were then cross-verified by another author (AD). Any discrepancies were resolved through group discussion. Corresponding authors for the papers [21,22,23,24] were contacted with requests for more information; unfortunately, we were unable to obtain answers to the questions we sought.

### 2.5. Quality Assessment

**Figure 1:**
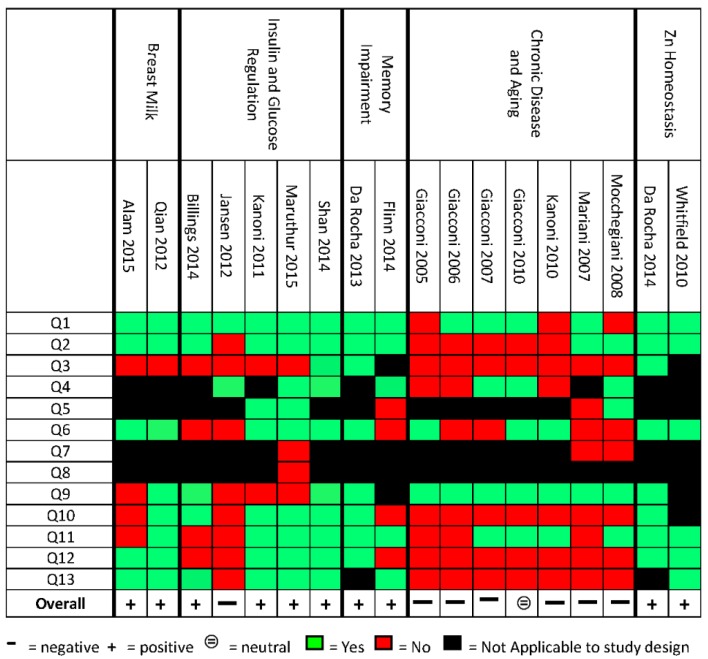
Quality Assessment (QA) results for each included study. Q1. Research question; Q2. Study Design; Q3. Subjects/patients bias; Q4. Group comparisons; Q5. Intervention/therapeutic description; Q6. Outcomes; Q7. Withdrawals; Q8. Blinding; Q9. Hardy-Weinberg equilibrium; Q10. Association between Zn intake or Zn status and Single Nucleotide Polymorphisms (SNPs); Q11. Appropriateness of statistics; Q12. Supported conclusions; Q13. Funding bias [29].

A suitable, validated quality assessment (QA) tool was not available for the types of studies and outcomes reviewed in this study. Therefore, an original quality assessment tool was created by merging and adapting the American Dietetic Association (ADA) quality criteria checklist for Randomized Control Trials (RCTs) and cohorts [25], the Cochrane risk of bias [26], and the STROBE statement checklist for observational studies [27] (Figure 1). The key domain that was included and considered particularly pertinent for this review was “polymorphisms are with the Hardy-Weinberg equilibrium” [28] and whether the association of the genetic variants with specific phenotypic traits was properly reported. The final checklist included 13 “yes” or “no” statements. Similar to the ADA quality criteria checklist and Cochrane risk of bias tool, each study was assigned as positive, neutral, or negative based on the number of “yes”, “no”, or “not applicable” answers. A study was assigned positive if the majority of statements received “yes”, or were assigned negative if the majority of statements received “no”. If a study had the same number of yes and no statements it was assigned neutral (Figure 1). Two reviewers (KD, MA, and AD) independently performed the quality assessment and any discrepancies were discussed as a group.

## 3. Results

### 3.1. Description of Included Studies

**Figure 2:**
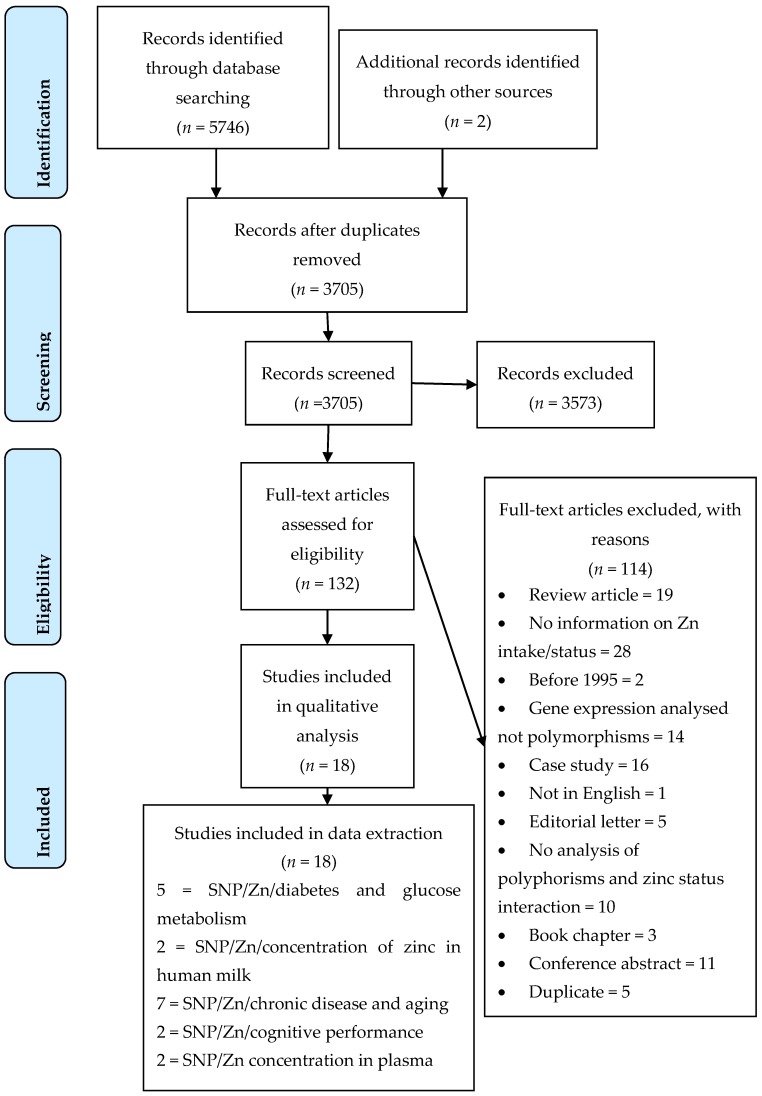
PRISMA 2009 Flow Diagram adapted from [44].

The result of the systematic search of the databases is shown in the PRISMA flowchart (Figure 2); eighteen studies matched the inclusion and exclusion criteria. Of these 18 studies, eight were observational cross-sectional [21,30,31,32,33,34,35], six were case control studies [23,36,37,38,39,40,41], three were non-randomised clinical control trials ([24,42,43], and one was a cross-sectional meta-analysis [22]. Nine separate genes with 31 SNPs (Table 1), and one study that looked at twins [34] were identified across the 18 studies. The studies selected were grouped according to the association between one or more polymorphisms with a phenotypic trait. Using this criterion, the studies were classified into the following five groups: polymorphisms in genes relating to Zn transporters for breastmilk, insulin and glucose regulation, cognitive performance, chronic disease in the ageing population, and Zn homeostasis (Table 1). 

The 18 studies were scored for their quality against a list of 13 items; a summary of the results of the quality assessment is summarised in Figure 1. Overall, the studies scored positively on the identification of the research questions and validation of methods; the studies identified as scoring negatively did so mainly in the selection of study participants, or providing conclusions that did not take into consideration the limitations of the study or study bias. Due to the lack of consistency across grouped studies, identification of outcomes, and methods of determining associations, conclusions on how to translate the information into recommendations in practice could not be drawn.

### 3.2. Does Dietary Zn Modulate the Effect of SLC30A2 Polymorphisms on Human Milk Zinc Content?

Two cross-sectional studies examined the association of polymorphisms within *SLC30A2* gene and Zn content variability in human milk from a total of 794 healthy, breastfeeding mothers with children from single births, without complications. Zn concentrations in breastmilk were examined at 42 days and 4 months postpartum [28]. *SLC30A2* SNPs found to be associated with variability in milk Zn concentrations are listed in Table 1. SNP rs-numbers were only reported in Qian et al. [30], whereas SNPs were identified as “novel” without associated rs-numbers in Alam et al. [28], which makes identifying common SNPs between the studies difficult. Qian et al. identified five SNPs in the Chinese mothers, with two associated with decreased concentrations and three with no association with Zn in breastmilk. In the US, Alam et al. identified 12 polymorphisms in mothers, of which two variants were associated with decreased concentrations of Zn in breastmilk. Low concentration of Zn in breastmilk were defined as 21.5 µmol/L [30], and less than 1 mg per L (15.3 µmol/L) [28]. Neither study reported whether maternal dietary Zn intake or plasma Zn modulated Zn concentrations of milk in mothers carrying polymorphisms associated with low breastmilk Zn concentrations [28,30]. Both of these studies were assessed by QA rating P (Figure 1).

### 3.3. Does Dietary Zn Modulate the Association between Gene Variants and Glucose Metabolism Traits in Relation to Type-2 Diabetes?

Five independent studies examined the association between SNPs for the *SLC30A8* gene, Zn intake, the risk of T2D diabetes, and glucose metabolism biomarkers (Table 1). Three out of five studies examined the SNP rs13266634 [36,41,43]; of these studies, all but Jansen et al. [36] confirmed the increased risk of T2D associated with the T allele of this SNP (Table 1). While Jansen and colleagues showed contradicting results, the small sample size may have not allowed the association to be significant, and this was discussed by the authors. When the effect of dietary Zn intake was investigated in carriers of the T allele for the SNP rs13266634, Shan et al. [41] reported a reversed risk for T2D with high plasma Zn concentration (third highest tertile ≥ 197.58 µg/dL). Maruthur et al. also reported that after Zn supplementation for 14 days carriers of the T allele experienced increased insulin response to glucose by 15% and 14% at 5 and 10 minutes, respectively, compared with individuals carrying the CC genotype [43]. 

The hypothesis that more variants of SLC30A8 are associated with impaired glucose metabolism was explored by Billings and co-workers in a study that sequenced the exome of the gene in 380 subjects classified as possessing an increased risk of developing T2D [21]. This study identified 44 novel *SLC30A8* variants; four of which demonstrated a positive association, and one that showed a negative association with pancreatic β-cell function biomarkers (Table 1). The authors concluded that dietary Zn intake did not modify the genetic predisposition to T2D, suggesting a limited role for dietary manipulation in affecting risk in relation to the SNPs identified. The study’s main limitation was a lack of description of a method to measure dietary intake which prevented the results from properly supporting the conclusion (Table 1 and Figure 1). The authors were contacted on this matter, however, we did not obtain a response.

The fifth study was a cross-sectional meta-analysis on 14 cohorts including a total of 46,021 individuals of European ancestry with fasting glucose <6 mmol/L [22]. The association of dietary zinc intake with fasting glucose, and the interaction with 20 genetic variants known to be related to glucose metabolism traits was examined. This study identified the *SLC30A8* SNP rs11558471, where carriers of the A allele have increased fasting glucose. An association was observed with the SNP rs11558471, fasting glucose, and total Zn intake, suggesting that Zn intake has an inverse association with fasting glucose plasma concentration in carriers of the A allele for that SNP. Fasting glucose of individuals carrying the A allele of rs11558471 responded to increased Zn intake compared with GG genotype carriers; for each milligram of Zn intake per day, a reduction of −0.0017 mmol/L fasting glucose was reported (Table 1) [22]. Quality of the meta-analysis was assessed as positive, however, the rationale for examining the specific SNP was not reported.

### 3.4. Does Dietary Zn Modulate the Effect of Gene Variants on Cognitive Performance?

Two studies, one in humans and one in mice, were identified that examined Zn, genes, and cognition. The human study examined the interaction between SNPs for the *SLC30A3* gene, cognitive performance, and Zn dietary intake in 240 healthy people over 50 years of age, but with memory deficit [32]. The authors reported a gene-nutrient interaction for SLC30A3 rs73924411 and serum Zn concentration (Table 1). Carriers of the T allele displayed higher memory score than the CC genotype carriers when serum Zn concentration was below the recommended concentration (<0.70 mg/L = 0.01 mmol/L). This suggests a possible neurotoxicity effect of Zn for T allele carriers with serum Zn > 0.70 mg/L = 0.01 mmol/L. 

The mouse model study examined late-onset AD, and analysed the interaction between Zn dietary intake and the isoform ε4 of the apolipoprotein E (ApoE) human gene [37] corresponding to SNP rs7412 in the NCBI database. The authors tested whether high Zn intake worsened the spatial memory of mice in which the endogen APOE gene was replaced with the isoform ε4 of human ApoE. The ApoE isoform ε4 is over-represented in AD patients and promotes the binding of Zn to amyloid plaques [45]. Although in a mouse model, Flinn’s group showed that high Zn intake was associated with worse performance in tests aimed at evaluating spatial memory in carriers of the ApoE isoform ε4 [37]. However, the authors did not report actual Zn intake, as the mice were allowed to drink Zn-spiked water ad-libitum.

Both these studies reported that high Zn intake, in association with specific gene variants, significantly impaired the memory scores of older adults and spatial memory in an AD mouse model, respectively. Flinn’s study scored negative in the QA assessment mainly because the lack of reporting of Zn intake. Da Rocha et al., although with some limitations, was assessed as positive in the QA (Figure 1) [32,37].

### 3.5. Does Dietary Zn Modulate the Association between Gene Variants and the Development of Chronic Diseases in the Aging Population?

Seven studies aimed to identify carriers of genetic polymorphisms that would benefit from Zn supplementation to support healthy ageing [23,24,35,38,39,40,46]. Unlike the other studies reported in this review, Zn and healthy ageing have not been associated with Zn transporter genes, but rather genes that are associated with inflammation and oxidative stress. Three papers investigated the association between Interleukin-6 (IL-6) SNP rs1800795 and serum Zn in a group of older adults, but did not show any conclusive relationship (Table 1) [24,35,46]. All papers in this group reported on SNPs in relation to separate outcomes, often in small sample sizes. Differing targets and the lack of consistency in reporting across the group prevents reaching a clinically relevant conclusion (Table 1) [23,35,38,39,40,42]. These studies were all assessed negative or neutral in the QA score (Figure 1).

### 3.6. Do Polymorphisms in Genes Involved in Zn Transport and Metabolism Affect Plasma Zn Concentration?

Two of the 18 papers identified did not explore the interaction between polymorphisms in Zn metabolism and dietary Zn in relation to a clinical biomarker/disease state, but they investigated the influence of polymorphisms only on plasma Zn concentrations and whether this is affected by dietary Zn intake [33,34]. Da Rocha et al. looked at the influence of SNPs rs11126936 and rs73924411 in the gene *SLC20A3* and found that Zn serum concentrations were significantly lower in carriers of C allele of rs11126936 compared to T carriers (*p* = 0.014). When subjects were grouped by serum Zn concentrations the CC genotype was more frequently observed in subjects with low Zn serum. Da Rocha and colleagues did not find any association between serum Zn concentration and rs73924411. This finding needs to be evaluated against another study from the same group that found a significant association between rs73924411 and Zn concentrations in relation to cognitive impairment scores [32]. This suggests the interaction is only observed in relation to cognitive impairment. Finally, Whitfield et al. [34] investigated to what extent Zn concentrations in erythrocytes are influenced by genetic effects by obtaining samples from twins. The group used model-fitting and grouped twins according to zygosity in order to establish to what extent the Zn concentration variation was due to genetic or environmental factors including diet. They concluded that 20% of the variation in Zn concentration is due to genetic factors [34].

## 4. Discussion

The completion of the Human Genome Project held the promise of resolving the complexity of individual response to diet and one-size fits all public health guidelines, and to reveal the role of genetics in shaping nutritional requirements [1,19]. Zn exemplifies this paradigm; this essential micronutrient is distributed throughout all organs, Zn absorption and metabolism involves numerous gene products, and it is expected that genetic variability is capable of affecting requirements [6]. 

The Zn transporter *SLC30A8* is a gene expressed in insulin secreting pancreas β-cells; *SLC30A8* SNP rs13266634 has been associated with increased risk of T2D [31,47,48,49,50]. The discovery of a relationship between Zn transporter gene variants and T2D led to the hypothesis that Zn intake may affect insulin and/or glucose metabolism [51], thus making *SLC30A8* polymorphisms good candidates to modify Zn requirements. This systematic review highlighted the role of Zn intake and its interaction with *SLC30A8* SNPs. In the meta-analysis performed by Kanoni et al. 2011, the gene dependent nutrient interaction with glucose metabolism markers was confirmed. Kanoni et al. demonstrated that total Zn intake has a stronger inverse association with fasting glucose concentration in individuals carrying the glucose-raising A allele of rs11558471. The interaction resulted in a reduction of 0.024 mmol/L in blood sugar concentration per 1 mg of Zn. The normal range for blood glucose concentration is considered 4–8 mmol/L; this finding, while interesting, may not be easily utilised in clinical practice due to the relatively small effects [22]. *SLC30A8* has been linked to pancreatic islets function [12]**,** but this was the first time that rs11558471 was investigated in the context of glucose metabolism outcomes. rs11558471 was reported to be in strong linkage disequilibrium with rs13266634, although no further explanation was offered on why this SNP was selected. Different SNPs on *SLC30A8* were tested by Shan’s group, showing that the risk of T2D and impaired glucose regulation could be attenuated by increasing plasma Zn concentrations in CC carriers of rs13266634. These observations support the concept that Zn intervention could play a role in T2D, and that Zn recommendations may benefit from being personalised according to *SLC30A8* genotypes. This study was assessed positive on quality, however, its use in clinical practice is limited, as it does not provide indications on the magnitude of an intervention (i.e., within or above the RDI) [41]. Jansen and collaborators reported rs13266634 in the same gene and presented conflicting results, reporting a lack of association with glucose metabolism biomarkers, possibly due to the small sample analysed [36]. This highlights the importance of further larger scale research projects that seek to clarify any gene-nutrient interactions and provide clear understanding of any intervention requirements.

The potential associations of genetic polymorphisms with cognitive performance in relation to Zn intake were investigated in two of the selected studies [32,37]. The *SLC30A3* gene was identified in 1996 and raised major interest, as the corresponding protein was shown to transport Zn into pre-synaptic vesicles of glutamatergic neurones of the cerebral cortex and hippocampus, key regions for the memory formation and with a role in the development of amyloid β plaques in AD [52,53]. Da Rocha and colleagues analysed the role of *SLC30A3* rs73924411 in memory [32]. Another study analysed another genetic variation associated with AD, the ApoE isoform ε4 [54]. Both reports suggested that carriers of T allele of *SLC30A3* rs73924411 and ApoE isoform ε4 (rs7412 in NCBI), respectively, could benefit from Zn plasma concentration on the lower side of the recommended cut-off and suggested that what is considered the optimal concentration could have a neurotoxic effect for the carriers of these polymorphisms. Those observations can lead to the conclusion that individuals with these genotypes would be better off in terms of cognitive performance with Zn intakes lower than the recommended amounts. Although one of these reports analysed a small sample [32] and the other did not report Zn intake correctly [54], they are examples of where one size fits all nutrient recommendations may not be appropriate. The quest to slow down the inevitable process of aging and the development of diseases associated with it has included the attempt to optimise diet to support the metabolic needs of elderly people. Seven papers indicated that there may be a potential interaction between markers of chronic disease in the elderly and the variation of Zn intake, with outcomes being dependent on genetic variations. However, these findings should be treated with caution until further research that explicitly quantifies the association of Zn intake and genotype, not just Zn status, is conducted. Therefore, no conclusions can be drawn at this time in relation to clinical practice. 

## 5. Perspectives

Healthcare professionals need to be able to translate genetic information in the context of the health priorities of patients, to take into consideration the scenario of a patient carrying one or more SNPs in genes affecting Zn metabolism in different organs and the possibility of the results of these different genotypes modifying the risk of two pathologies in opposite/different ways. This would ideally require bioinformatics tools to evaluate the function and effect size of each relevant variant, along with clinical information. To develop dietary recommendations that incorporate information of common polymorphisms, more studies need to be conducted looking at the association of genetic variants, disease biomarkers, and dietary intake. The majority of the studies included in this report analysed the association of only two out of three of these factors, making it difficult to draw a conclusion about personalised dietary recommendations based on genotypes. Moreover, a reliable biomarker to assess Zn status is still an open debate; serum concentration is generally used, but this measure is affected by several transient factors, such as infections and acute inflammation [55].

A number of studies identified in this review confirmed the concept of genetic makeup modifying the response to Zn and possibly Zn requirements; however, we are still left with a number of questions such as what is the optimal Zn intake for specific genetic variations? Should the advice on Zn intake change in relation to a reduction in disease risk or to be used as a therapeutic tool to make treatments more effective? Most studies did not assess dietary Zn intake, so while associations between Zn status and SNPs may have been identified, there is no mention of whether current dietary recommendations are appropriate for different genotypes for general good health or in relation to disease outcomes. Another point of reflection is that most studies were performed on small cohorts, in specific sub groups of the population, not representing the variability present in the human species. These questions are important for research in the current environment, where more nutrigenetic tests are being developed and advertised as tools to tailor dietary advice; the public have increasing access to these tests through both a range of healthcare professionals and the internet. While tests may correctly report on the associations between genetics and health effects, they do not take into consideration the effect size and the direction of the intervention. The limited clear evidence on how variations affect dietary requirements for general good health, therapeutic treatments, and disease reduction, raises the question of deciding when it is useful to use the results to modify nutrient intake advice. Healthcare professionals with appropriate knowledge in both genetics and nutrition are required to help individuals understand whether and how information within nutrigenetic tests should be used to inform dietary intake. Limitations of this systematic study include the lack of homogeneity between the studies selected, which prevented the possibility of performing a meta-analysis, thereby making it difficult to reach a decisive conclusion that provides clear directions for application. 

## Figures and Tables

**Table 1:** Data extraction of each study identified. T1D, type 1 diabetes; T2D, type 2 diabetes.

Study Reference	Study Design	Sample Description	SNPs Associated with a Phenotype (Gene, rs# and Nucleotide Change)	Description of the SNPs Association with Phenotypic Trait	SNPs Association with Plasma Zn Concentration ^	SNPs Association with Zn Intake or Zn Supplementation	Outcomes
**Polymorphisms in Zn transporters for Breast milk**							
Alam et al. 2015 [30]	Cross-sectional	54 F, healthy American **Inclusion**: 18–40 years who were breastfeeding one healthy infant at ≈4 months post-partum. **Exclusion**: Pre-term births (<37 week gestation), multiple births, smokers	12 novel SNPs identified as missense by sequencing *SLC30A2* gene: A^28^D, K^66^N, Q^71^H, D^103^E, A^105^P Q^117^H, T^288^S, A^310^T, L^311^V, T^312^K, V^313^G, Q^315^R.	D^103^E found associated with low [Zn] breastmilk.	N/A	N/A	Variability in the concentration of Zn in human milk was associated with *SLC30A2* SNPs. Possibly because of the small sample size, the other association did not reach significance.
Qian et al. 2012 [31]	Cross-sectional	750 F, healthy Chinese **Inclusion**: 18–36 years breastfeeding day 42 postpartum. **Exclusion**: multiple gestations, multiparity, pre-existing maternal diseases, foetal malformation and lactation failure, complicated pregnancy	1 SNP in the promoter region and 1 in the coding region of the coding sequence of *SLC30A2* gene (rs117153535 (G/T); No rs-*SLC30A2*/1031A > G)	Association between genetic polymorphisms and milk Zn concentration: rs117153535 T allele associated with lower [Zn]; *SLC30A2*/1031A > G allele associated with lower [Zn].	N/A	N/A	Variability in the concentration of Zn in human milk was associated with *SLC30A2* SNPs.
**Polymorphisms in genes relating to Insulin and Glucose Regulation**							
Billings et al. 2014 [21]	Cross-sectional from RCT	2997 male and female participants in the Diabetes Prevention Program (US) **Inclusion**: high risk of developing T2D (overweight with elevated fasting glucose and IGT) randomised to placebo, metformin 850 mg twice daily, or lifestyle intervention and consented to genetic testing	44 novel SNPs including: *SLC30A8*: rs2464591 (C/T), rs2466296 (C/T), rs2466297 (A/G), rs2466299 (C/T)	Associated with improvement in β-cell function.	No association between Zn intake and SNPs on diabetes incidence	N/A	*SLC30A8* variants influence T2D risk, insulin secretion traits and β-cell function. Zn intake did not modify the genetic risk. None of the SNPs had a large effect.
			SLC30A8: rs2466293 (C/T)	Decrease in β-cell function.			
Shan et al. 2014 [41]	Cross-sectional Case-control study	1796 male and female Chinese Han ethnicity **Inclusion**: newly diagnosed IGR and T2D, ≥30 years, BMI ≥ 40 kg/m^2^, no history of diabetes diagnosis or pharmacological treatment for hyperlipidaemia. **Exclusion**: clinically significant neurological, endocrine, or other systemic diseases, as well as acute illness, chronic inflammatory, or infectious diseases.	*SLC308A*, rs13266634 (C/T)	C allele was associated with increased odds of T2D.	Decreased risk of T2D in carriers of risk (C) allele with high plasma Zn concentration.	N/A	*SLC30A8* rs13266634 is associated with T2D. CC genotype and low plasma Zn increase T2D risk and Impaired Glucose Regulation. High plasma Zn concentration (third highest tertile ≥197.58 µg/dL) decreased risk of T2D in carriers of risk C allele.
Kanoni et al. 2011 [22]	Cross-sectional Meta-analysis	Meta-analysis from 14 cohort studies, 46,021 participants. **Inclusion**: no diabetes (fasting glucose ≥ 7 mmol/L or use of antidiabetic medications). Sample size for interaction analysis between dietary Zn intake and SNPs ranged from 27,010 to 45,821.	20 SNPs analysed including: *SLC30A8*, rs11558471 (A/G)	N/A	Zn intake of 14 mg/day associated with 0.024 mmol/L decrease in fasting glucose concentration in A carriers in comparison to GG (0.048 mmol/L reduction for AA homozygotes)	Interaction only significant with zinc diet and supplementation	Increased Zn intake improves diabetes risk dependent for A allele carriers of SNP rs11558471.S
Jansen et al. 2012 [36]	Case-control study	150 male and female (22 T1D; 53 T2D; 7 matched controls). **Inclusion**: ≥18 years, patients with type 1 or 2 diabetes; **Exclusion**: acute infection, recent, cancer, liver or renal disease.	*SLC30A8* rs13266634 (C/T) MT1A rs11640851 (A/C), rs8052394 (A/G)	No significance in SNP association T1D or T2D	N/A	N/A	Serum Zn decreased in patients with diabetes, no association with any SNPs and disease biomarkers. Small sample size.
Maruthur et al. 2015 [43]	Non-randomised supplement clinical trial	55 male and female Old Order Amish from Lancaster, PA, USA. **Inclusion**: 21–70 years no diagnosis of diabetes and random glucose less than 11.10 mmol/L [200 mg/dL].	*SLC30A8,* rs13266634 (C/T) (R^325^W). The (C) allele encodes the arginine (R), and the (T) allele encodes the tryptophan (W)	Carriers of allele T showed increased insulin response after supplementation	Serum Zn concentration increased by 23% for CC genotypes and 33% for CT/TT genotypes after supplementation	Oral Zn acetate 50 mg, twice daily × 14 days	Carriers of risk T allele showed increased insulin response after supplementation with Zn; these participants may benefit most from Zn supplementation
**Polymorphisms in genes relating to Memory Impairment**							
Da Rocha et al. 2014a [32]	Cross-sectional study	240 male and female mature, elderly adults **Inclusion**: ≥50 years, absence of dementia, owning intellect enough to continue production, present complaint of progressive memory loss, show objective evidence of memory deficits. **Exclusion**: symptoms of depression, anxiety, or stress, IQ < 70, use of vitamin supplements containing micronutrients of interest	SLC30A3, rs73924411 (C/T) rs11126936 (G/T)	Increased frequency of rs11126936 TT carriers in people with memory deficits.	When serum Zn was below recommended concentration: rs11126936 T carriers had better memory scores, whereas CC carriers’ performance decreased.	N/A	Recommended Zn concentration may be neurotoxic for T carriers suffering from memory deficit. CC genotype may benefit from increased Zn intake when Zn serum is low
Flinn et al. 2014 [37]	Case-control on mouse-model	22 Mouse strain CRND8 with human ApoE ε4; 23 Mouse strain CRND8; 24 WT controls	ApoE ε4 (Apoliprotein E isoform SNP in NCBI rs7412)	Carriers of human ApoE ε4 showed impaired spatial memory	N/A	10 ppm of ZnCO_3_ in water	Increased dietary Zn significantly impaired spatial memory of mice carrying ApoE ε4 human compared with WT and CRND8. The amount of Zn consumed daily was not reported
**Polymorphisms in genes relating to chronic disease in the aging population**							
Giacconi et al. 2007 [40]	Case-control study	506 male and female elderly adults (288 with coronary artery stenosis; 218 healthy, age and sex matched controls living at home) **Inclusion**: older individuals born and living in Central Italy admitted to INRCA Geriatric Hospital, Ancona, Italy, for endarterectomy	*MT2A* rs10636 (C/G)	C allele more frequent amongst patients with carotid stenosis	Low plasma [Zn] associated with C allele	N/A	*MT2A* rs10636 C allele is associated with low plasma Zn and independently with carotid stenosis. No conclusive evidence that modifying Zn intake is beneficial for C allele carriers
Kanoni et al. 2010 [35]	Cross-sectional study	819 male and female elderly adults (272 from Italy, 163 from Greece, 137 from Germany, 128 from France, and 119 from Poland); **Inclusion**: (ZINCAGE Project: www.zincage.org). ≥ 60 years old, non-institutionalised, free of medication and supplements. **Exclusion**: autoimmune, neurodegenerative, cardiovascular, kidney, or liver disease, diabetes, infections, cancer, sickle cell, skin ulcerations, and endocrine disorders.	−174 IL-6 G/C (inNational Center for Biotechnology Information (NCBI)rs1800795 C/G)	GG genotype had greater increase in IL-6 levels with increased ‘Zn diet score’ than CG and CC genotypes	Significant interaction of ‘Zn diet score’ and GG genotype of rs1800795	N/A	Method developed to calculate Zn dietary intake and correlate it with plasma Zn “Zn score”. Association observed between Zn score, G allele and IL-6 levels
Giacconi et al. 2005 [38]	Case-control study	279 male and female born and living in Central Italy, (91 T2D; 188 age and sex-matched controls living at home). **Inclusion**: (**ZINCAGE Project: www.zincage.org**) older adults, diagnosis of T2D with carotid stenosis, control individuals living at home, no hypertension, diabetes or carotid stenosis, no history of Coronary Heart Disease (CHD,) normal electrocardiography and no sign of myocardial ischemia	*MT2A* rs1610216 A/G (in NCBI database C/T)	AA genotype associated with carotid stenosis and T2D, inflammation markers. A allele more frequent in patients than controls	Plasma Zn concentration decreased in AA genotype compared with AG genotypes	N/A	*MT2A* rs1610216, AA genotype associated with disease biomarkers and low serum Zn concentration. No evidence supporting that increased serum Zn would be beneficial
Giacconi et al. 2010 [39]	Case-control study	459 male and female elderly Italian adults (215 Cardio-Vascular Diseases (CVD,) 244 age and sex-matched healthy controls); 374 male and female elderly Greek adults (154 CVD 220 age and sex-matched healthy controls). **Inclusion**: (**ZINCAGE Project: www.zincage.org**) Italian: diagnosis of ischemic heart disease and/or carotid heart disease; Greek: diagnoses history of angina, heart failure, coronary heart disease, stroke, myocardial infarction, or heart surgery. **Exclusion**: Italian controls: diabetes diagnosis	*MT1A* rs8052394 (A/C), rs11640851 (A/G)	Increased frequency of rs11640851 G allele carriers in Greek patients with ischemic heart and/or carotid heart disease in comparison to Greek controls. No difference between Italian patients and controls.	Intracellular zinc of peripheral blood cells decreased in CVD patients *MT1A* haplotype CG+ compared with *MT1A* CG−/CG− haplotype	N/A	No evidence of association between genotype, Zn, and biomarkers of disease, including inflammation and circulating lipids
Giacconi et al. 2006 [23]	Case-control study	406 male and female older adults born and living in central Italy (105 with carotid stenosis and CVD (C); 111 with carotid stenosis, no symptoms or cardioischaemia (D); 190 age and sex-matched controls). **Inclusion**: ≥70 years, older individuals born and living in Central Italy admitted to INRCA Geriatric Hospital, Ancona, Italy, for endarterectomy	1267 *Hsp70-2* (A/G); (in NCBI database rs780016316 C/T) -308 *TNFα* G/A (in NCBI rs1800629 A/G)	1267 *Hsp70-2* G allele more frequent in (C) group than (D)	Plasma Zn similar across genotypic groups	N/A	No evidence of association between genotype, Zn, and biomarkers of disease, including hypertension and circulating lipids.
Mocchegiani et al. 2008 [42]	Non-randomised supplement clinical trial	110 male and female healthy, non-institutionalised older adults from Italy, France, Germany, Poland, and Greece. **Inclusion**: (ZINCAGE Project: www.zincage.org). 60–84 years old, free of medication, plasma zinc ≤ 10.5 μM. **Exclusion**: autoimmune, neurodegenerative, cardiovascular, kidney or liver diseases, diabetes, infections, cancer, chronic inflammatory bowel disease or acrodermatitis enteropathica, sickle cell anaemia, chronic skin ulcerations, and endocrine disorders	−174 *IL-6* G/C (in NCBI rs1800795 C/G)	No significant difference between carriers of alleles for immune parameters	GG genotype had significantly lower plasma Zn than C carriers. GG genotypes with normal plasma Zn still presented with impaired Zn status	10 mg/day Zn-aspartate for 48 ± 2 days	Low plasma Zn associated with impaired immune response and psychological function independent of genotype GG genotype carriers are more predisposed to Zn deficiency and suggested as better candidates for supplementation
Mariani et al. 2008 [24]	Non-randomised supplement clinical trial	39 male and female healthy older adults **Inclusion**: (ZINCAGE Project: www.zincage.org). 60–83 years old, healthy old people, still living independently, plasma Zn < 11μmol/L. **Exclusion**: taking medication, nutritional integrators, or vitamin complexes.	+647 A/C *MT1A* −174 *IL-6* (in NCBI rs1800795 (C/G)	+647 *MT1A* genotype associated with increased inflammatory biomarkers	+647 *MT1A* C− allele associated with lower plasma Zn than C+ at basal and after supplementation.	10 mg/day Zn-aspartate for 48 ± 2 days. Plasma Zn concentration significantly increased after supplementation in C+ carriers of −174 *IL6.*	Carriers of C- genotype of *MT1A* had lowest concentration of plasma zinc. Increment after supplementation was more pronounced in subjects carrying C- allele of *MT1A* /C+ −174 *IL-6*. Carries of C− *MT1A*/C −174 *IL-6* did not respond to zinc supplementation.
**Polymorphisms relating to Zn homeostasis**							
Whitfield et al. 2010 [34]	Cross-sectional study	2926 male and female adult twins living in Australia **Inclusion**: Born between 1903 and 1964 (30–92 years old), enrolled in the Australian Twin Registry	N/A	N/A	20% of the variation in plasma Zn concentration is due to genetic factors.	N/A	Genetic variability is contributing factor to Zn plasma variability. Specific genotypes not reported
Da Rocha et al. 2014b [33]	Cross-sectional study	110 male and female older adults **Inclusion**: ≥50 years old adults **Exclusion**: use of vitamin supplements containing micronutrients	*SLC30A3* rs11126936 (A/C)	N/A	CC genotypes had lower plasma Zn concentration than A carriers. CC genotype more frequent in participants with low plasma Zn	N/A	*SLC30A3* polymorphism rs11126936 was associated with differences in plasma Zn concentration

## Supplementary Materials

The following are available online at http://www.mdpi.com/2072-6643/9/2/148/s1, Figure S1: Databases’ search strategy, Figure S2: PRISMA checklist.
