# Supplementary material for: Genetic Variations as Modifying Factors to Dietary Zinc Requirements—A Systematic Review
Source: Nutrients. 2017 Feb 17;9(2):148. doi: 10.3390/nu9020148 (PMC5331579; doi:10.3390/nu9020148)
Supplement: Supplementary file 1 [file nutrients-09-00148-s001.zip › Database search S1.pdf]

Web of Science search:

1. TS=(Zinc OR Zn)
2. TS=(Finger*)
3. #1 NOT #2
4. TS=(Polymorph* OR Allel* OR Genet* OR Geno* OR Gene OR Genes)
5. TS=(Zinc NEAR/2 deficienc*)
6. TS=(Zn NEAR/2 deficienc*)
7. TS=(Zinc NEAR/2 transporter*)
8. TS=(Zn NEAR/2 transporter*)
9. #5 OR #6 OR #7 OR #8
10. #3 AND #4 AND #9

[excluding] **RESEARCH AREAS:** (NUCLEAR SCIENCE TECHNOLOGY OR PLANT SCIENCES OR VETERINARY SCIENCES OR MICROSCOPY OR AGRICULTURE OR INFECTIOUS DISEASES OR MATHEMATICAL COMPUTATIONAL BIOLOGY OR MICROBIOLOGY OR ZOOLOGY OR INSTRUMENTS INSTRUMENTATION OR GEOCHEMISTRY GEOPHYSICS OR MARINE FRESHWATER BIOLOGY OR FORESTRY OR ENGINEERING OR EVOLUTIONARY BIOLOGY OR VIROLOGY OR CHEMISTRY OR EDUCATION EDUCATIONAL RESEARCH OR ENVIRONMENTAL SCIENCES ECOLOGY OR PARASITOLOGY OR SPECTROSCOPY OR FISHERIES OR PUBLIC ADMINISTRATION OR FOOD SCIENCE TECHNOLOGY OR PHYSICS OR MINERALOGY OR WATER RESOURCES OR MYCOLOGY OR MATERIALS SCIENCE OR GEOLOGY OR BEHAVIORAL SCIENCES OR COMPUTER SCIENCE OR BUSINESS ECONOMICS OR PSYCHOLOGY OR ANTHROPOLOGY)

OVID MEDLINE, CINAHL and EMBASE search:

1. Zinc.ti,ab.
2. Zn.ti,ab.
3. Zinc/ (subject heading)
4. Zinc compounds/
5. Finger*.ti,ab.
6. 1 OR 2 OR 3 OR 4
7. 6 NOT 7
8. (Polymorph* OR Allel* OR Genet* OR Geno* OR Gene OR Genes).ti,ab.
9. Polymorphism, Genetic/
10. Exp Polymorphism, Genetic/
11. 8 OR 9 OR 10
12. Exp Cation Transport Proteins/
13. (Zinc adj2 deficienc*).ti,ab.
14. (Zn adj2 deficienc*).ti,ab.
15. (Zinc adj2 transporter*).ti,ab.
16. (Zn adj2 transporter*).ti,ab.
17. 13 OR 14 OR 15 OR 16
18. 7 AND 11 AND 12 AND 17

(Scopus search):

1. TITLE-ABS-KEY(Zinc OR Zn)
2. TITLE-ABS-KEY(finger*)
3. #1 AND NOT #2
4. TITLE-ABS-KEY(polymorph* OR allel* OR genet* OR gene OR geno* OR genes)
5. TITLE-ABS-KEY(Zinc W/2 deficienc*)
6. TITLE-ABS-KEY(Zn W/2 deficienc*)

7. TITLE-ABS-KEY(Zinc W/2 transporter*)
8. TITLE-ABS-KEY(Zn W/2 transporter*)
9. #5 OR #6 OR #7 OR #8
10. #3 AND #4 AND #9

(EXCLUDE (SUBJAREA , "AGRI") OR EXCLUDE (SUBJAREA , "ENVI") OR EXCLUDE (SUBJAREA , " MATE") OR EXCLUDE (SUBJAREA , "VETE") OR EXCLUDE (SUBJAREA , "CENG") OR EXCLUDE (S UBJAREA , "COMP") OR EXCLUDE (SUBJAREA , "SOCI") OR EXCLUDE (SUBJAREA , "EART") OR EXCLUDE (SUBJAREA , "ENGI") OR EXCLUDE (SUBJAREA , "PHYS") OR EXCLUDE (SUBJAREA , "A RTS") OR EXCLUDE (SUBJAREA , "MATH"))

(1547 RESULTS ON 12/10/15)
